# The association between child maltreatment and health risk behaviours and conditions throughout life in the Australian Child Maltreatment Study

**DOI:** 10.5694/mja2.51877

**Published:** 2023-04-02

**Authors:** David M Lawrence, Anna Hunt, Ben Mathews, Divna M Haslam, Eva Malacova, Michael P Dunne, Holly E Erskine, Daryl J Higgins, David Finkelhor, Rosana Pacella, Franziska Meinck, Hannah J Thomas, James G Scott

**Affiliations:** ^1^ Curtin University Perth WA; ^2^ Queensland University of Technology Brisbane QLD; ^3^ Bloomberg School of Public Health Johns Hopkins University Baltimore MD United States of America; ^4^ The University of Queensland Brisbane QLD; ^5^ QIMR Berghofer Medical Research Institute Brisbane QLD; ^6^ Institute for Community Health Research Hue University Hue City Vietnam; ^7^ Queensland Centre for Mental Health Research Brisbane QLD; ^8^ Institute of Child Protection Studies Australian Catholic University Melbourne VIC; ^9^ Crimes against Children Research Center University of New Hampshire Durham NH United States of America; ^10^ Institute for Lifecourse Development University of Greenwich London United Kingdom; ^11^ University of Edinburgh Edinburgh United Kingdom; ^12^ University of the Witwatersrand Johannesburg Johannesburg South Africa

**Keywords:** Healthcare disparities, Health status indicators, Child abuse, Child welfare

## Abstract

**Objective:**

To estimate associations between all five types of child maltreatment (emotional abuse, neglect, physical abuse, sexual abuse, and exposure to domestic violence) and health risk behaviours and conditions.

**Design, setting, participants:**

Nationally representative survey of Australian residents aged 16 years and older conducted by computer‐assisted telephone interviewing.

**Main outcome measures:**

Associations between child maltreatment and the following health risk behaviours and conditions: current smoker, binge drinking (at least weekly in past 12 months), cannabis dependence (according to the Cannabis Severity of Dependence Scale), obesity (based on body mass index), self‐harm in past 12 months, and suicide attempt in past 12 months.

**Results:**

A total of 8503 participants completed the survey. All five types of child maltreatment were associated with increased rates of all of the health risk behaviours and conditions that we considered. The strongest associations were in the youngest age group (16–24‐year‐olds). Sexual abuse and emotional abuse were associated with the highest odds of health risk behaviours and conditions. Cannabis dependence, self‐harm and suicide attempts were most strongly associated with child maltreatment. Experiencing more than one type of child maltreatment was associated with higher rates of health risk behaviours and conditions than experiencing one type of child maltreatment.

**Conclusions:**

Child maltreatment is associated with substantially increased rates of health risk behaviours and conditions. Prevention and intervention efforts should be informed by trauma histories, and holistic psychosocial care should be incorporated into programs focusing on behaviour change.

**The known:** Globally, childhood maltreatment is associated with diverse negative outcomes in childhood and across the lifespan, including a range of health risk behaviours and conditions.

**The new:** The Australian Child Maltreatment Study has shown that the majority of Australians have experienced child maltreatment, and that these experiences are associated with substantially higher rates of health risk behaviours and conditions, including substance misuse, self‐harm and suicide attempts. Sexual abuse and emotional abuse present the highest risks.

**The implications:** Trauma‐informed health promotion strategies and interventions aimed at preventing health risk behaviours and conditions may require holistic psychosocial interventions.

To our knowledge, the Australian Child Maltreatment Study (ACMS) has produced the first national estimates of prevalence of child maltreatment (emotional abuse, neglect, physical abuse, sexual abuse, and exposure to domestic violence). As reported elsewhere in this supplement, child maltreatment is a common experience in Australia.^1^, ^2^ About two‐thirds of Australian adults reported experiencing at least one of the five types of child maltreatment before the age of 18 years,^1^ and more than half of them experienced more than one type of child maltreatment.^2^

Child maltreatment is associated with many negative health and social outcomes across the lifespan.^3^, ^4^, ^5^, ^6^ The impact of child maltreatment is pervasive across many mental disorders and physical illnesses. This includes maltreatment being associated with elevated lifetime risk of cancer; cardiovascular, respiratory and genitourinary diseases; depression and anxiety; post‐traumatic stress disorder; substance misuse disorders; and self‐harm and suicide.^4^, ^5^ Pathways from child maltreatment to these negative outcomes are complex. One of the most direct ways in which maltreatment causes harm is through its effect on health risk behaviours and conditions, many of which emerge early in the life course and persist for decades.

Several theoretical mechanisms have been posited for causal associations between child maltreatment and subsequent health risks.^7^ First, the distress associated with child maltreatment can be long lasting and can prompt maladaptive coping mechanisms such as substance misuse.^8^, ^9^, ^10^, ^11^ Second, unresolved trauma is associated with emotional numbing, and victim‐survivors may self‐harm to seek feeling.^3^, ^6^ Third, the trauma associated with child maltreatment can be so pervasive that victim‐survivors feel both thwarted belongingness and perceived burdensomeness.^12^, ^13^ With experience of emotional numbing and self‐harming behaviour, victim‐survivors of child maltreatment may also acquire capability for suicide.^6^, ^13^, ^14^

In this article, we report the prevalence of six health risk behaviours and conditions: smoking, binge drinking, cannabis dependence, obesity, self‐harm, and suicide attempts. We also examine how the prevalence of each of these health risks is associated with each of the five types of child maltreatment, and how associations between child maltreatment and health risks vary by age group and gender. As most of the hypothesised mechanisms for the impact of child maltreatment on subsequent health risks are associated with emotional and behavioural rather than physical pathways, we hypothesise that health risk behaviours and conditions are more common in people who have experienced child maltreatment, and that non‐physical maltreatment is as harmful as physical and sexual abuse.

## Methods

### Participants

Full details of the ACMS methodology are provided elsewhere in this supplement.^15^ Briefly, ACMS participants were recruited by random digit dialling of mobile phones; each potential participant was sent an advance text message, and this was followed up with a verbal invitation to participate by an interviewer. Demographic distribution of the sample was compared with 2016 census data, and data were weighted to adjust for higher representation of Australian born and higher socio‐economic status participants. Comparison with Australian census and 2017 survey data showed that the weighted data were representative of the population of Australians aged 16 years and older.^15^

### Measures

Child maltreatment was assessed using the Juvenile Victimisation Questionnaire‐R2 adapted version (Australian Child Maltreatment Study). The 16 screener items measured all five types of child maltreatment (emotional abuse, neglect, physical abuse, sexual abuse, and exposure to domestic violence). The survey also asked about health risk behaviours and conditions, which included: cigarette smoking in the past 12 months; binge drinking (having six or more drinks for men or five or more drinks for women in a single session at least weekly over the past 12 months); cannabis dependence (Cannabis Severity of Dependence Scale score of 3 or more);^16^, ^17^ obesity (body mass index > 30 kg/m^2^ based on self‐reported height and weight); non‐suicidal self‐injury (answering yes to the question “during the past 12 months have you deliberately harmed or injured yourself, without intending to end your own life?”); and suicide attempt (answering yes to the question “during the past 12 months, have you attempted suicide?”). Except for the Cannabis Severity of Dependence Scale item, these items were drawn from the 2007 Australian National Survey of Mental Health and Wellbeing.^18^

### Statistical analysis

Experiences of physical abuse, sexual abuse and exposure to domestic violence were based on positive endorsement of any of the screener items for these child maltreatment types, regardless of how many times the experience happened. Conceptually, emotional abuse and neglect require a pattern of behaviour, so we only counted positive endorsements to the screener items if the experience occurred over a period of weeks, months or years; this meant that participants who reported a duration of days for emotional abuse and neglect were classified as non‐maltreated for these two types.^1^

Respondents could refuse to answer any question they were uncomfortable with. The amount of missing data was less than 1% for each type of child maltreatment and each health risk behaviour and condition that we considered. While some participants for whom such data were missing may have experienced child maltreatment, we have conservatively chosen to treat missing data as non‐endorsements. As such, our prevalence estimates for child maltreatment and health risks may be slightly underestimated.

Survey‐weighted prevalence of each health risk behaviour or condition was calculated by experience of child maltreatment (any maltreatment, each type of maltreatment, subtypes of physical abuse and sexual abuse, and number of maltreatment types [none, one, two, and more than two]) and by gender and age group, and 95% confidence intervals were calculated using the method of expansion in Taylor series.^19^ Logistic regression models were fitted for each of the six health risk behaviours and conditions. For each health risk, three separate models were fitted: experience of any child maltreatment compared with no child maltreatment; separately for each of the five types of maltreatment using a three‐level indicator (experienced that type of maltreatment, experienced any other type of maltreatment, no child maltreatment); and all five types of child maltreatment fitted simultaneously as independent binary (yes/no) variables. Each model was fitted with two levels of adjustment for other factors: adjusted for age group, gender and child maltreatment only (simple adjustment), and adjusted for age group, gender, child maltreatment, socio‐economic status (based on postcode of residence and quintiles of the Index of Relative Socio‐Economic Disadvantage [one of the Socio‐Economic Indexes for Areas]),^20^ experience of financial hardship during childhood, and current level of financial strain (fully adjusted).

All analyses were conducted using SAS 9.4. To ensure quality, two of us (EM and DH) randomly spot‐checked the SAS coding and results in SPSS 27.

### Ethics approval

The Queensland University of Technology Human Research Ethics Committee approved the study (1900000477).

## Results

A total of 8503 participants completed the survey. All six health risks were more common in those who experienced child maltreatment compared with those who did not (Box 1). Estimated prevalence rates were significantly higher for people who experienced child maltreatment for all health risks in women (*P* < 0.05) and all health risks except obesity in men (*P* < 0.05) (Box 2).

Box 1 **Prevalence of health risk behaviours and conditions, by experience of child maltreatment**

**Table:** 

	Number (percentage) of participants*		
	Did not experience any child maltreatment (*n* = 3223)	Experienced any child maltreatment (*n* = 5280)	Total sample (*N* = 8503)
Current smoker	316 (11.1%)	996 (21.1%)	1312 (17.3%)
Binge drinking^†^	271 (8.4%)	597 (12.6%)	868 (11.0%)
Cannabis dependence	21 (0.5%)	238 (3.7%)	259 (2.5%)
Obesity^‡^	580 (24.4%)	1214 (28.2%)	1794 (26.8%)
Self‐harm in past 12 months	47 (0.7%)	365 (4.7%)	412 (3.2%)
Suicide attempt in past 12 months	12 (0.3%)	122 (1.5%)	134 (1.1%)

* Participant numbers are unweighted sample counts and percentages are weighted prevalence estimates.

† Six or more drinks (men) or five or more drinks (women) at least weekly in past 12 months.

‡ In the obese body mass index range (> 30 kg/m^2^); as 379 participants did not know or refused to provide their weight and/or height, prevalence estimates for obesity were calculated by excluding these participants.

Box 2 Proportions of adults who reported health risk behaviours and conditions, by experience of child maltreatment and gender*

**Figure:**
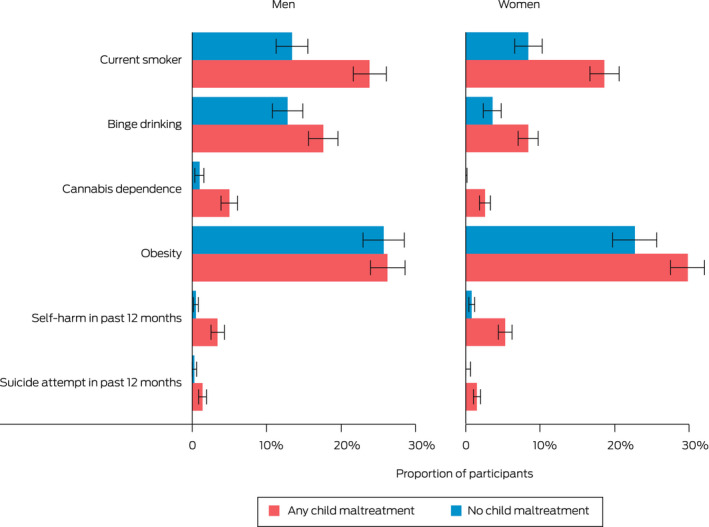
* Bar lengths represent estimated proportion of the population and error bars represent 95% CIs.

The differences between prevalence rates for people who experienced child maltreatment versus those who did not were highest for lower frequency health risk behaviours and conditions. As an example of one of the lower frequency health risks, an estimated 196 900 people who experienced child maltreatment had attempted suicide in the 12 months before the survey compared with 22 300 people who did not (rates of 1.5% *v* 0.3%). As examples of relatively high frequency health risks, about 608 300 people who experienced child maltreatment had self‐harmed in the 12 months before the survey compared with 52 100 people who did not (4.7% *v* 0.7%), and about 481 100 people who experienced child maltreatment were classified as having cannabis dependence compared with 42 400 who did not (3.7% *v* 0.5%). Rates were similar across each of the five types of child maltreatment (Box 3).

Box 3 Proportions of adults who reported health risk behaviours and conditions, by experience of child maltreatment, age group and gender*

**Figure:**
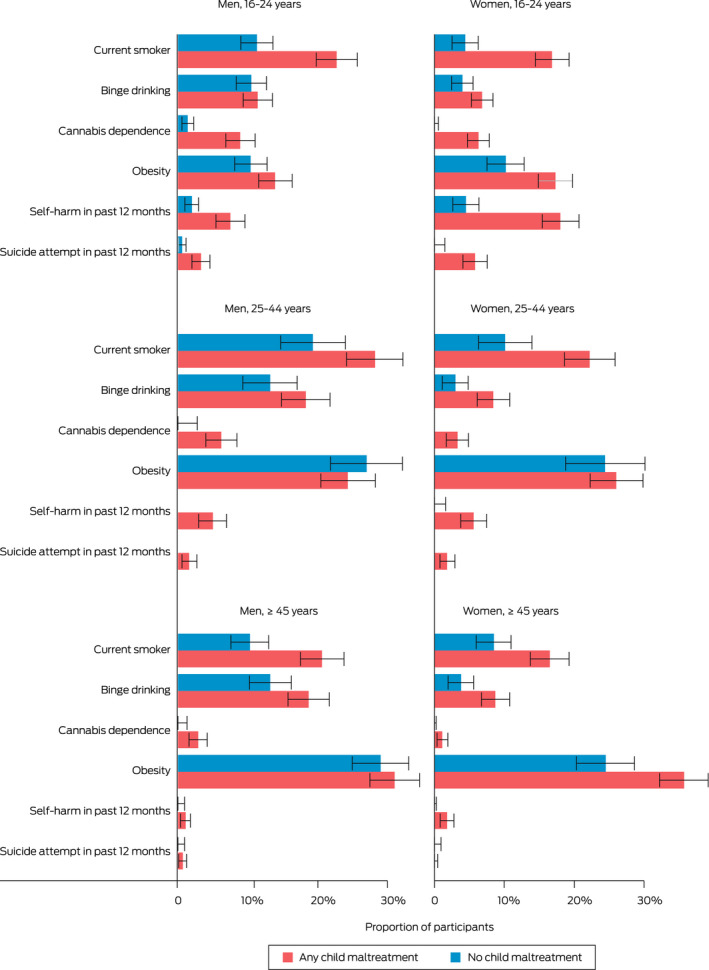
* Bar lengths represent estimated proportion of the population and error bars represent 95% CIs.

Health risk behaviours and conditions were more common in people who had experienced multiple types of child maltreatment. For instance, 831 200 of those who experienced one type of child maltreatment were current smokers (17.6%) compared with 1 882 200 of those who experienced two or more types of maltreatment (23.1%), a statistically significant difference. This pattern was also statistically significant for cannabis dependence, self‐harm and suicide attempts (Supporting Information, table 1).

Rates of health risk behaviours and conditions varied across age groups; for example, current smoking was highest in 25–44‐year‐olds. However, the differences in proportions of current smokers between those who had and had not experienced child maltreatment were higher for 16–24‐year‐olds and those aged ≥ 45 years (Box 3).

Binge drinking was more common in men than women. For men the prevalence was higher in the middle and older age groups. The differences in proportions of those who reported binge drinking between those who had and had not experienced child maltreatment were larger for women in the middle and older age groups (Box 3). The prevalence of cannabis dependence was much lower than that for cigarette smoking or binge drinking, but it was strongly associated with child maltreatment. There were no participants aged 25 years or older with no experience of child maltreatment who were dependent on cannabis (Box 3).

Rates of obesity only varied between those with and without experience of child maltreatment for women aged 16–24 years and women aged ≥ 45 years, for whom substantial differences were observed (Box 3).

Rates of self‐harm and suicide attempts varied significantly between people with and without experience of child maltreatment. Rates of self‐harm and suicide attempts were highest for 16–24‐year‐olds. In the middle and older age groups, self‐harm and suicide attempts were only reported in those who had experienced child maltreatment. In the middle and older age groups, the estimated prevalence of self‐harm was about zero in both men and women without experience of child maltreatment (Box 3).

We used logistic regression to examine the relationships between child maltreatment and health risk behaviours and conditions. Considering any experience of child maltreatment compared with no child maltreatment, all health risks were significantly elevated. Also, after adjusting for age group, gender, socio‐economic status, financial hardship during childhood and current financial strain, all health risks were significantly elevated in those who experienced child maltreatment. In addition, for most estimates, the attenuation of odds ratios was relatively modest when controlling for socio‐economic factors (odds ratio reduced from 7.13 to 6.18 for cannabis dependence, from 6.72 to 3.93 for self‐harm, and from 5.12 to 4.56 for suicide attempt; Box 4).

Box 4 Odds ratios for health risk behaviours and conditions for adults who experienced child maltreatment relative to those who did not*

**Table:** 

	Simply adjusted odds ratio (95% CI)^†^	Fully adjusted odds ratio (95% CI)^‡^
Current smoker	2.08 (1.76–2.47)	1.87 (1.62–2.16)
Binge drinking	1.63 (1.35–1.98)	1.31 (1.12–1.54)
Cannabis dependence	7.13 (3.79–13.4)	6.18 (3.92–9.76)
Obesity	1.20 (1.04–1.38)	1.17 (1.04–1.32)
Self‐harm in past 12 months	6.72 (4.50–10.2)	3.93 (2.86–5.41)
Suicide attempt in past 12 months	5.12 (2.20–11.8)	4.56 (2.46–8.43)

* All odds ratios were significantly higher than 1 at *P* < 0.05.

† Model adjusted for age group and gender only.

‡ Model adjusted for age group, gender, socio‐economic status (based on postcode of residence and quintiles of the Index of Relative Socio‐Economic Disadvantage), experience of financial hardship during childhood, and current financial strain.

Considering the five types of child maltreatment, we modelled associations with health risks in two ways. First, we considered each type of maltreatment one at a time with a binary (yes/no) indicator, while adjusting for experience of any other type of child maltreatment with a binary indicator. Using this approach, all five types of child maltreatment were significantly associated with higher odds of all six health risks, when adjusting for age group and gender only, and all except for the association between neglect and binge drinking remained significant when adjusting for socio‐economic factors (Supporting Information, table 2). Second, we modelled all five types of child maltreatment simultaneously as independent binary variables. Using this approach, sexual abuse was independently associated with higher odds of all six health risks, and emotional abuse was independently associated with higher odds of all health risks except binge drinking. Also, exposure to domestic violence was associated with higher odds of current smoking and higher odds of cannabis dependence, and physical abuse was associated with higher odds of suicide attempt (Supporting Information, table 3).

Additional data from the study relating to the association between experience of child maltreatment and health risks by age and sex are presented in the Supporting Information (table 4 and figures 1–6).

## Discussion

In our survey, Australians who had experienced child maltreatment were more likely to report all six health risk behaviours and conditions. Considered individually, all five types of child maltreatment were associated with higher rates of health risks. When considered jointly, emotional abuse and sexual abuse were both independently associated with higher rates of health risks. Health risks were significantly more common in people who experienced multiple types of child maltreatment. Although all six health risk behaviours and conditions that we examined are known to be associated with socio‐economic status,^21^, ^22^ our adjusted models showed that associations between child maltreatment and health risks were only modestly attenuated after adjustment for socio‐economic factors.

While several studies have reported associations between health risk behaviours and conditions and adverse child experiences in adolescents,^3^, ^6^, ^9^, ^10^ there is limited information on associations between specific types of child maltreatment and health risks across the life course. While sexual abuse is a well known risk factor for adverse life outcomes including health risks,^9^, ^14^, ^21^ our data show that emotional abuse was as strongly associated with each health risk as sexual abuse. Moreover, sexual abuse and emotional abuse were the two maltreatment types with the highest odds ratios for multiple health risks, being especially strongly associated with suicide attempts, self‐harm, cannabis dependence, smoking and binge drinking (Supporting Information, table 3). A birth cohort study has found a similar association between emotional abuse in childhood and mental health outcomes at age 30 years,^23^ and it is possible that mental health problems associated with child maltreatment are an important pathway to these health risks.

In adults who had no history of child maltreatment, prevalence of cannabis dependence, self‐harm in the previous 12 months and suicide attempts in the previous 12 months were almost zero. While the ACMS is a cross‐sectional study, child maltreatment was assessed up to age 18 years, and all six health risks that we considered were current or had occurred in the previous 12 months. This suggests that these harmful behaviours and conditions persist well beyond the experience of child maltreatment. While a cross‐sectional study cannot demonstrate cause and effect, the strong associations and negligible rates among older adults with no experience of child maltreatment are consistent with possible causal association. While less prevalent overall, cannabis dependence, self‐harm and suicide attempts had substantially higher odds ratios for associations with health risks than smoking, binge drinking and obesity, suggesting that there may be few causal mechanisms associated with these health risks that do not involve experience of trauma or child maltreatment.

Strong associations between each of the five types of child maltreatment and cigarette smoking and binge drinking were found across the three age groups. Child maltreatment may lead to onset of substance misuse in adolescence, and ongoing dependence on substances throughout life.^8^, ^9^, ^10^ The persistence of these maladaptive coping strategies in older age groups extends the harms caused by childhood trauma by contributing to poor health in older age.^24^, ^25^

These findings have implications for the design of health promotion interventions. Many interventions to reduce health risk behaviours and conditions, particularly substance misuse and obesity, are focused on the specific health risk with little consideration of comorbid issues such as mental health, family dysfunction and experience of trauma.^26^, ^27^ The effectiveness of interventions to reduce health risks may be enhanced by incorporating program design measures to address trauma histories. Holistic psychosocial care rather than siloed behavioural interventions are likely to be more appropriate and effective.^27^, ^28^

### Strengths and limitations

To our knowledge, the ACMS is the first nationally representative study of the prevalence and impacts of child maltreatment in Australia. Its large size and rigorous assessment of child maltreatment provide statistical power that is sufficient to assess the associations between child maltreatment and health risk behaviours and conditions throughout life. Although we have investigated associations between child maltreatment and recent health risk behaviours and conditions, our data cannot be used to determine causal pathways. Also, due to questionnaire length constraints, we could not assess other health risks that have been associated with child maltreatment in other studies, including misuse of substances other than cannabis and alcohol and risky sexual behaviour. As noted elsewhere in this supplement, few participants identified as gender diverse.^16^ For this reason, and because of small cell sizes and heterogeneity and other important aspects of gender‐diverse identification, detailed analysis of findings for these participants will be reported separately (manuscript in preparation).

### Conclusion

Health risk behaviours and conditions are common, and child maltreatment was associated with higher rates of all health risks considered in this study. Interventions to address these health risks need to consider the role of childhood trauma in health risk onset and persistence — for example, explicit recognition of the need for and benefit of more adaptive coping mechanisms for dealing with past trauma, and incorporation of an explicit focus on the association between mental health and health risk behaviours. Prevention of child maltreatment may provide enormous benefits in terms of improving the wellbeing of children and the health the Australian population during adolescence and adulthood.

## Open access

Open access publishing facilitated by Curtin University, as part of the Wiley ‐ Curtin University agreement via the Council of Australian University Librarians.

## Agency roles

The NHMRC funded the ACMS. The Australian Government and the Australian Institute of Criminology provided supplementary funding for several specific questions. The researchers were independent from the funders.

## Competing interests

No relevant disclosures

## Supporting information


**Supporting Information**.
