# Supplementary material for: The association between child maltreatment and health risk behaviours and conditions throughout life in the Australian Child Maltreatment Study
Source: Med J Aust. 2023 Apr 2;218(Suppl 6):S34–9. doi: 10.5694/mja2.51877 (PMC10952518; doi:10.5694/mja2.51877)
Supplement: Supplementary file 1 — Supporting Information. [file MJA2-218-S34-s001.pdf]

The Medical Journal of Australia

Supporting Information

Supplementary results

This appendix was part of the submitted manuscript and has been peer reviewed. It is posted as supplied by the authors.

Appendix to: Lawrence DM, Hunt A, Mathews B, et al. The association between child maltreatment and health risk behaviours and conditions throughout life in the Australian Child Maltreatment Study. *Med J Aust* 2023; doi: 10.5694/mja2.51877.

Supporting Information

Table 1. Proportions of adults with health risk behaviours and conditions, by experience of child maltreatment and gender

	Percentage (95% CI)		
	**Men**	**Women**	**All genders***
**Current smokers**			
No child maltreatment	13.4% (11.3–15.5%)	8.4% (6.6–10.3%)	11.1% (9.6–12.5%)
Any child maltreatment	23.8% (21.6–26.0%)	18.6% (16.7–20.6%)	21.1% (19.6–22.6%)
Emotional abuse	26.5% (23.2–29.9%)	21.1% (18.4–23.9%)	23.4% (21.3–25.5%)
Neglect	30.2% (23.1–37.2%)	23.0% (17.7–28.4%)	26.1% (21.9–30.3%)
Physical abuse	24.7% (21.7–27.7%)	20.2% (17.4–23.1%)	22.5% (20.5–24.6%)
Sexual abuse	26.7% (22.7–30.7%)	21.7% (19.0–24.4%)	23.5% (21.3–25.7%)
Exposure to domestic violence	25.8% (23.1–28.6%)	18.2% (15.8–20.6%)	21.9% (20.1–23.7%)
One type of maltreatment	20.0% (16.7–23.4%)	15.2% (11.9–18.5%)	17.6% (15.3–19.9%)
Two types of maltreatment	24.5% (20.2–28.8%)	17.5% (13.7–21.4%)	21.1% (18.2–24.0%)
More than two types of maltreatment	28.1% (24.1–32.2%)	22.0% (18.9–25.1%)	24.5% (22.1–27.0%)
**Binge drinking**			
No child maltreatment	12.8% (10.8–14.9%)	3.6% (2.4–4.8%)	8.4% (7.2–9.7%)
Any child maltreatment	17.6% (15.6–19.6%)	8.4% (7.0–9.7%)	12.6% (11.4–13.8%)
Emotional abuse	17.7% (14.8–20.7%)	9.2% (7.3–11.1%)	12.6% (10.9–14.2%)
Neglect	16.7% (10.9–22.6%)	6.8% (4.0–9.6%)	10.7% (7.9–13.5%)
Physical abuse	17.4% (14.8–20.1%)	8.5% (6.6–10.4%)	12.9% (11.3–14.6%)
Sexual abuse	20.2% (16.5–23.8%)	9.4% (7.5–11.3%)	13.0% (11.3–14.8%)
Exposure to domestic violence	18.0% (15.5–20.4%)	8.1% (6.4–9.7%)	12.7% (11.3–14.1%)
One type of maltreatment	19.2% (15.9–22.5%)	7.2% (5.0–9.4%)	13.2% (11.2–15.2%)
Two types of maltreatment	14.0% (10.7–17.4%)	8.9% (6.1–11.7%)	11.6% (9.4–13.8%)
More than two types of maltreatment	18.7% (15.2–22.3%)	9.0% (6.9–11.1%)	12.7% (10.8–14.6%)
**Cannabis dependence**			
No child maltreatment	1.0% (0.4–1.6%)	np	0.5% (0.2–0.9%)
Any child maltreatment	5.0% (3.9–6.1%)	2.6% (1.9–3.3%)	3.7% (3.1–4.4%)
Emotional abuse	6.9% (5.0–8.8%)	3.3% (2.3–4.3%)	4.8% (3.8–5.7%)
Neglect	7.6% (3.8–11.4%)	3.9% (2.0–5.8%)	5.3% (3.5–7.2%)
Physical abuse	6.2% (4.6–7.9%)	2.5% (1.4–3.5%)	4.5% (3.5–5.4%)
Sexual abuse	6.9% (4.6–9.3%)	3.2% (2.2–4.2%)	4.6% (3.5–5.6%)
Exposure to domestic violence	5.4% (4.1–6.8%)	3.0% (2.0–4.0%)	4.2% (3.4–5.0%)
One type of maltreatment	3.2% (1.6–4.7%)	1.8% (0.6–2.9%)	2.4% (1.5–3.4%)
Two types of maltreatment	4.4% (2.4–6.3%)	1.9% (0.5–3.3%)	3.3% (2.1–4.6%)
More than two types of maltreatment	7.9% (5.6–10.2%)	3.6% (2.4–4.9%)	5.3% (4.1–6.5%)
**Obesity**			
No child maltreatment	25.7% (22.9–28.5%)	22.7% (19.7–25.6%)	24.4% (22.4–26.4%)
Any child maltreatment	26.2% (23.9–28.6%)	29.8% (27.5–32.0%)	28.2% (26.6–29.8%)
Emotional abuse	30.6% (26.9–34.3%)	31.3% (28.2–34.3%)	31.1% (28.7–33.4%)
Neglect	28.8% (21.7–35.8%)	38.2% (32.0–44.4%)	34.9% (30.2–39.5%)
Physical abuse	28.9% (25.6–32.1%)	32.6% (29.3–36.0%)	30.7% (28.4–33.0%)
Sexual abuse	27.2% (23.2–31.3%)	33.1% (29.9–36.2%)	31.4% (28.9–33.8%)
Exposure to domestic violence	26.5% (23.6–29.4%)	29.7% (26.9–32.6%)	28.3% (26.3–30.3%)
One type of maltreatment	21.6% (18.3–24.9%)	27.6% (23.7–31.5%)	24.7% (22.2–27.3%)
Two types of maltreatment	28.1% (23.5–32.7%)	25.8% (21.4–30.1%)	26.9% (23.8–30.1%)
More than two types of maltreatment	30.5% (26.2–34.8%)	33.7% (30.1–37.3%)	32.5% (29.8–35.2%)
**Self-harm in past 12 months**			
No child maltreatment	0.5% (0.2–0.8%)	0.8% (0.4–1.2%)	0.7% (0.4–0.9%)
Any child maltreatment	3.4% (2.5–4.3%)	5.3% (4.4–6.2%)	4.7% (4.1–5.4%)
Emotional abuse	4.4% (2.9–5.8%)	7.6% (6.2–9.1%)	6.8% (5.8–7.9%)
Neglect	6.6% (3.1–10.1%)	8.3% (5.6–11.0%)	8.5% (6.3–10.7%)
Physical abuse	3.5% (2.4–4.6%)	5.7% (4.4–7.0%)	5.0% (4.1–5.9%)
Sexual abuse	5.3% (3.2–7.3%)	6.9% (5.5–8.3%)	6.9% (5.7–8.1%)
Exposure to domestic violence	3.9% (2.7–5.1%)	6.3% (5.0–7.5%)	5.5% (4.6–6.4%)
One type of maltreatment	2.2% (1.0–3.3%)	1.9% (1.0–2.8%)	2.1% (1.3–2.8%)
Two types of maltreatment	3.8% (1.9–5.8%)	5.2% (3.2–7.3%)	4.5% (3.2–5.9%)
More than two types of maltreatment	4.7% (3.0–6.4%)	8.1% (6.4–9.8%)	7.5% (6.2–8.7%)

**Suicide attempt in past 12 months**			
No child maltreatment	0.3% (0–0.6%)	np	0.3% (0.1–0.5%)
Any child maltreatment	1.4% (0.9–1.9%)	1.5% (1.0–2.0%)	1.5% (1.2–1.9%)
Emotional abuse	2.1% (1.1–3.0%)	2.3% (1.5–3.1%)	2.3% (1.7–2.9%)
Neglect	4.2% (1.2–7.3%)	2.7% (1.2–4.3%)	3.5% (2.0–5.0%)
Physical abuse	2.0% (1.2–2.9%)	1.7% (1.1–2.4%)	2.0% (1.4–2.5%)
Sexual abuse	2.2% (0.9–3.4%)	2.0% (1.3–2.7%)	2.2% (1.6–2.8%)
Exposure to domestic violence	1.6% (0.9–2.4%)	1.9% (1.2–2.5%)	1.9% (1.4–2.3%)
One type of maltreatment	0.6% (0–1.1%)	0.4% (0–0.8%)	0.5% (0.1–0.8%)
Two types of maltreatment	1.1% (0.2–2.0%)	1.0% (0.3–1.7%)	1.1% (0.5–1.6%)
More than two types of maltreatment	2.7% (1.4–4.1%)	2.7% (1.7–3.7%)	2.9% (2.1–3.7%)

np = not published because of small cell size but included in totals where applicable. * Includes participants who identified as non-binary, transgender or other, and those who did not state their gender.

Table 2. Odds ratios for health risk behaviours and conditions for adults who experienced any types of child maltreatment relative to those who did not, by type of maltreatment*

	Unadjusted odds ratio (95% CI)†	Fully adjusted odds ratio (95%CI)‡
**Current smoker**		
Emotional abuse	**2.37 (1.96–2.87)**	**2.11 (1.80–2.48)**
Neglect	**2.79 (2.14–3.65)**	**2.27 (1.79–2.88)**
Physical abuse	**2.21 (1.83–2.67)**	**2.04 (1.74–2.39)**
Sexual abuse	**2.61 (2.15–3.17)**	**2.38 (2.01–2.81)**
Exposure to domestic violence	**2.10 (1.76–2.52)**	**1.92 (1.64–2.24)**
**Binge drinking**		
Emotional abuse	**1.70 (1.36–2.13)**	**1.31 (1.09–1.58)**
Neglect	**1.49 (1.06–2.09)**	1.16 (0.86–1.57)
Physical abuse	**1.61 (1.29–2.00)**	**1.33 (1.11–1.60)**
Sexual abuse	**1.98 (1.57–2.48)**	**1.53 (1.26–1.85)**
Exposure to domestic violence	**1.61 (1.30–1.98)**	**1.29 (1.09–1.54)**
**Cannabis dependence**		
Emotional abuse	**9.21 (4.82–17.6)**	**8.14 (5.10–13.1)**
Neglect	**10.6 (5.20–21.5)**	**9.35 (5.36–16.3)**
Physical abuse	**8.50 (4.44–16.3)**	**6.98 (4.28–11.2)**
Sexual abuse	**10.7 (5.57–20.5)**	**8.63 (5.30–13.9)**
Exposure to domestic violence	**7.41 (3.91–14.0)**	**6.92 (4.33–11.0)**
**Obesity**		
Emotional abuse	**1.39 (1.18–1.63)**	**1.28 (1.11–1.47)**
Neglect	**1.68 (1.32–2.12)**	**1.30 (1.05–1.61)**
Physical abuse	**1.33 (1.14–1.56)**	**1.26 (1.11–1.45)**
Sexual abuse	**1.38 (1.17–1.62)**	**1.29 (1.12–1.49)**
Exposure to domestic violence	**1.22 (1.05–1.42)**	**1.14 (1.00–1.30)**
**Self-harm in past 12 months**		
Emotional abuse	**9.10 (5.97–13.9)**	**5.30 (3.71–7.38)**
Neglect	**10.9 (6.66–17.7)**	**6.14 (4.08–9.22)**
Physical abuse	**7.40 (4.82–11.4)**	**4.79 (3.42–6.71)**
Sexual abuse	**10.6 (6.86–16.4)**	**6.21 (4.42–8.73)**
Exposure to domestic violence	**7.28 (4.78–11.1)**	**4.02 (2.89–5.58)**
**Suicide attempt in past 12 months**		
Emotional abuse	**7.38 (3.20–17.0)**	**6.73 (3.45–12.6)**
Neglect	**10.8 (4.27–27.2)**	**8.19 (3.92–17.0)**
Physical abuse	**6.87 (2.92–16.2)**	**6.45 (3.22–12.3)**
Sexual abuse	**8.20 (3.36–20.0)**	**7.07 (3.70–13.5)**
Exposure to domestic violence	**5.74 (2.50–13.2)**	**4.91 (2.61–9.24)**

* Separate models fit for each type of sexual abuse with three categories of exposure: experienced this type of maltreatment during childhood, experienced any other type of maltreatment during childhood, did not experience maltreatment during childhood; bold text denotes that odds ratio is significantly higher than 1 at $P < 0.05$. † Model adjusted for age group and gender only. ‡ Model adjusted for age group, gender, socio-economic status (based on postcode of residence and quintiles of the Index of Relative Socio-Economic Disadvantage), experience of financial hardship during childhood, and current financial strain.

Table 3. Odds ratios for health risk behaviours and conditions for adults who experienced individual types of child maltreatment relative to those who did not, by type of maltreatment*

	Simple adjustment odds ratio (95% CI)†	Fully adjusted odds ratio (95% CI)‡
**Current smoker**		
Emotional abuse	**1.29 (1.06–1.58)**	**1.24 (1.06–1.45)**
Neglect	1.20 (0.92–1.56)	1.08 (0.87–1.35)
Physical abuse	1.14 (0.95–1.38)	1.19 (1.02–1.37)
Sexual abuse	**1.66 (1.39–1.98)**	**1.61 (1.39–1.85)**
Exposure to domestic violence	1.16 (0.96–1.40)	**1.19 (1.03–1.38)**
**Binge drinking**		
Emotional abuse	1.16 (0.91–1.48)	1.03 (0.86–1.25)
Neglect	0.82 (0.59–1.15)	0.84 (0.63–1.11)
Physical abuse	1.05 (0.84–1.31)	1.09 (0.91–1.29)
Sexual abuse	**1.55 (1.26–1.91)**	**1.35 (1.14–1.60)**
Exposure to domestic violence	1.13 (0.91–1.39)	1.07 (0.90–1.27)
**Cannabis dependence**		
Emotional abuse	**1.77 (1.11–2.81)**	**1.83 (1.29–2.61)**
Neglect	1.10 (0.69–1.75)	1.21 (0.84–1.75)
Physical abuse	1.49 (0.99–2.25)	1.20 (0.88–1.65)
Sexual abuse	**2.46 (1.61–3.75)**	**2.02 (1.51–2.71)**
Exposure to domestic violence	1.42 (0.87–2.30)	**1.87 (1.33–2.63)**
**Obesity**		
Emotional abuse	**1.19 (1.01–1.41)**	**1.16 (1.01–1.34)**
Neglect	**1.30 (1.02–1.65)**	1.05 (0.85–1.28)
Physical abuse	1.12 (0.96–1.30)	1.13 (0.99–1.29)
Sexual abuse	**1.22 (1.05–1.42)**	**1.18 (1.04–1.34)**
Exposure to domestic violence	0.95 (0.81–1.11)	0.92 (0.81–1.05)
**Self-harm in past 12 months**		
Emotional abuse	**2.36 (1.61–3.47)**	**2.06 (1.56–2.72)**
Neglect	1.21 (0.81–1.79)	1.21 (0.89–1.65)
Physical abuse	0.93 (0.65–1.32)	1.25 (0.97–1.60)
Sexual abuse	**3.07 (2.17–4.33)**	**2.68 (2.11–3.40)**
Exposure to domestic violence	1.44 (0.99–2.09)	1.11 (0.85–1.45)
**Suicide attempt in past 12 months**		
Emotional abuse	**2.09 (1.12–3.88)**	**2.31 (1.40–3.81)**
Neglect	1.61 (0.86–3.02)	1.36 (0.87–2.14)
Physical abuse	1.47 (0.79–2.75)	**1.78 (1.14–2.79)**
Sexual abuse	**2.56 (1.49–4.38)**	**2.25 (1.52–3.35)**
Exposure to domestic violence	1.29 (0.76–2.17)	1.12 (0.71–1.77)

* Single models fit with adjusted effects fitted for each type of child maltreatment; bold text denotes that odds ratio is significantly higher than 1 at $P < 0.05$. † Model adjusted for age group and gender only. ‡ Model adjusted for age group, gender, socio-economic status (based on postcode of residence and quintiles of the Index of Relative Socio-Economic Disadvantage), experience of financial hardship during childhood, and current financial strain.

Table 4. Proportions of adults with health risk behaviours or conditions, by experience of child maltreatment, age group and gender

	Percentage (95% CI)		
	**Men**	**Women**	**All genders***
**16–24-year-olds**			
Current smoker			
No child maltreatment	11.3% (9.0–13.6%)	4.4% (2.5–6.2%)	8.4% (6.8–9.9%)
Any child maltreatment	22.7% (19.8–25.7%)	16.8% (14.4–19.2%)	19.8% (17.9–21.6%)
Binge drinking			
No child maltreatment	10.5% (8.3–12.7%)	4.0% (2.4–5.5%)	7.6% (6.2–9.0%)
Any child maltreatment	11.4% (9.3–13.5%)	6.8% (5.3–8.4%)	8.8% (7.6–10.1%)
Cannabis dependence			
No child maltreatment	0.9% (0.2–1.5%)	np	0.6% (0.2–1.0%)
Any child maltreatment	6.6% (4.8–8.5%)	4.9% (3.5–6.2%)	5.9% (4.7–7.0%)
Obesity			
No child maltreatment	7.8% (5.9–9.8%)	8.4% (6.1–10.7%)	8.0% (6.6–9.5%)
Any child maltreatment	11.1% (9.0–13.2%)	15.3% (13.0–17.5%)	13.6% (12.1–15.2%)
Self-harm in past 12 months			
No child maltreatment	2.0% (1.0–2.9%)	4.5% (2.6–6.4%)	3.0% (2.1–4.0%)
Any child maltreatment	7.5% (5.4–9.6%)	18.0% (15.4–20.7%)	14.3% (12.6–16.0%)
Suicide attempt in past 12 months			
No child maltreatment	0.6% (0.1–1.2%)	np	0.6% (0.2–1.1%)
Any child maltreatment	3.3% (2.0–4.6%)	5.8% (4.1–7.5%)	5.2% (4.0–6.3%)
**25–44-year-olds**			
Current smoker			
No child maltreatment	19.3% (14.7–24.0%)	10.1% (6.3–13.9%)	15.2% (12.1–18.3%)
Any child maltreatment	28.2% (24.1–32.2%)	22.2% (18.6–25.8%)	25.1% (22.4–27.8%)
Binge drinking			
No child maltreatment	13.2% (9.3–17.1%)	3.0% (1.1–4.8%)	8.6% (6.3–11.0%)
Any child maltreatment	18.3% (14.8–21.8%)	8.4% (6.1–10.8%)	13.1% (11.1–15.2%)
Cannabis dependence			
No child maltreatment	np	np	np
Any child maltreatment	5.2% (3.1–7.3%)	2.5% (1.1–3.8%)	3.7% (2.5–4.9%)
Obesity			
No child maltreatment	23.2% (18.4–28.0%)	21.9% (16.6–27.3%)	22.9% (19.3–26.5%)
Any child maltreatment	21.6% (17.8–25.3%)	24.6% (21.0–28.3%)	23.6% (21.0–26.2%)
Self-harm in past 12 months			
No child maltreatment	np	np	np
Any child maltreatment	5.0% (3.0–6.9%)	5.6% (3.8–7.5%)	5.6% (4.2–7.0%)
Suicide attempt in past 12 months			
No child maltreatment	np	np	np
Any child maltreatment	1.6% (0.6–2.7%)	1.8% (0.8–2.9%)	1.7% (1.0–2.5%)
**≥ 45-year-olds**			
Current smoker			
No child maltreatment	10.3% (7.6–13.0%)	8.5% (6.0–11.0%)	9.4% (7.6–11.3%)
Any child maltreatment	20.6% (17.5–23.7%)	16.5% (13.7–19.3%)	18.4% (16.3–20.5%)
Binge drinking			
No child maltreatment	13.2% (10.2–16.2%)	3.8% (1.9–5.6%)	8.6% (6.8–10.3%)
Any child maltreatment	18.7% (15.7–21.7%)	8.7% (6.7–10.7%)	13.2% (11.5–15.0%)
Cannabis dependence			
No child maltreatment	np	np	np
Any child maltreatment	1.9% (0.9–2.9%)	0.9% (0.2–1.6%)	1.4% (0.8–2.1%)
Obesity			
No child maltreatment	26.5% (22.6–30.4%)	19.8% (16.1–23.5%)	23.2% (20.5–25.9%)
Any child maltreatment	27.1% (23.7–30.5%)	30.9% (27.6–34.1%)	29.1% (26.8–31.5%)
Self-harm in past 12 months			
No child maltreatment	np	np	np
Any child maltreatment	1.1% (0.4–1.8%)	1.8% (0.8–2.8%)	1.5% (0.8–2.1%)
Suicide attempt in past 12 months			
No child maltreatment	np	np	np
Any child maltreatment	0.7% (0.1–1.2%)	np	0.4% (0.1–0.7%)

np = not published because of small cell size but included in totals where applicable. * Includes participants who identified as non-binary, transgender or other, and those who did not state their gender.

Figure 1. Proportions of adults who were current smokers, by experience of child maltreatment and gender*

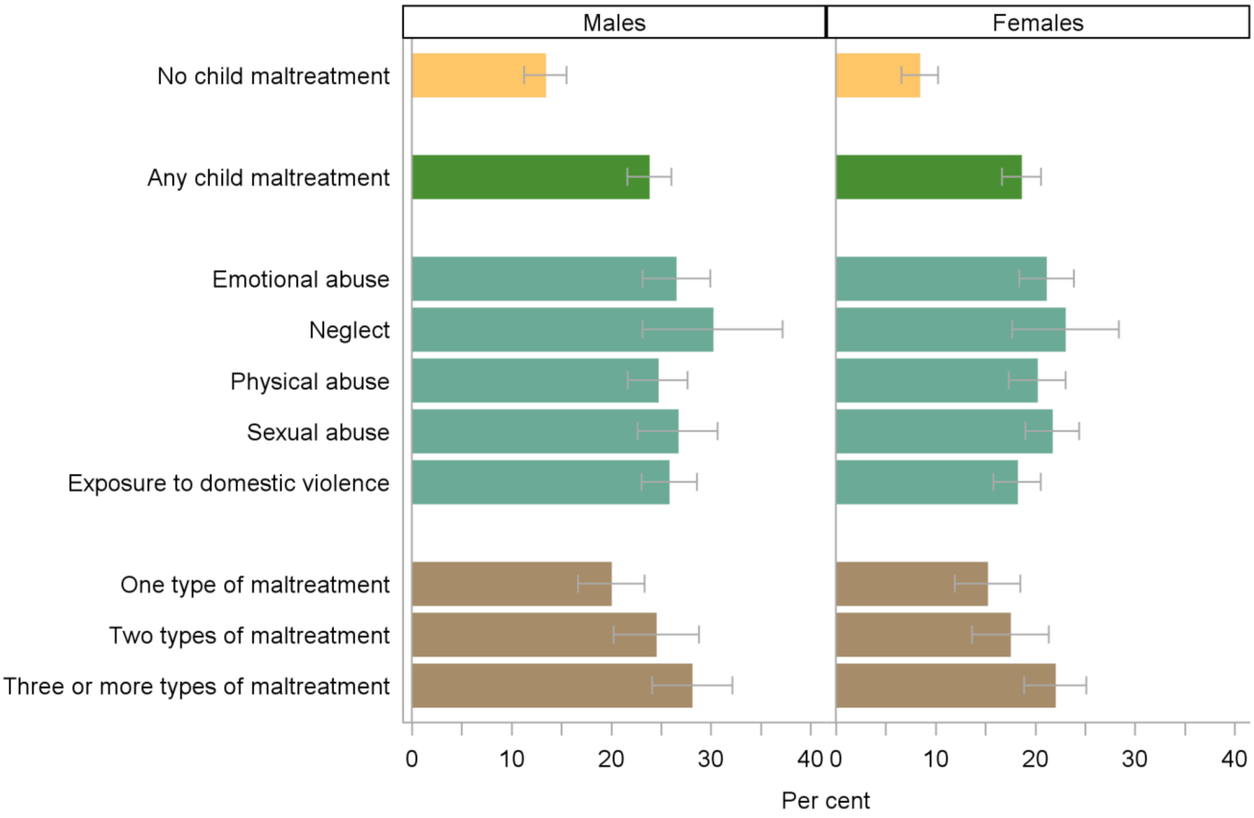

* Bar lengths represent estimated proportion of the population and error bars represent 95% CIs.

Figure 2. Proportions of adults who reported binge drinking weekly or more often, by experience of child maltreatment and gender*

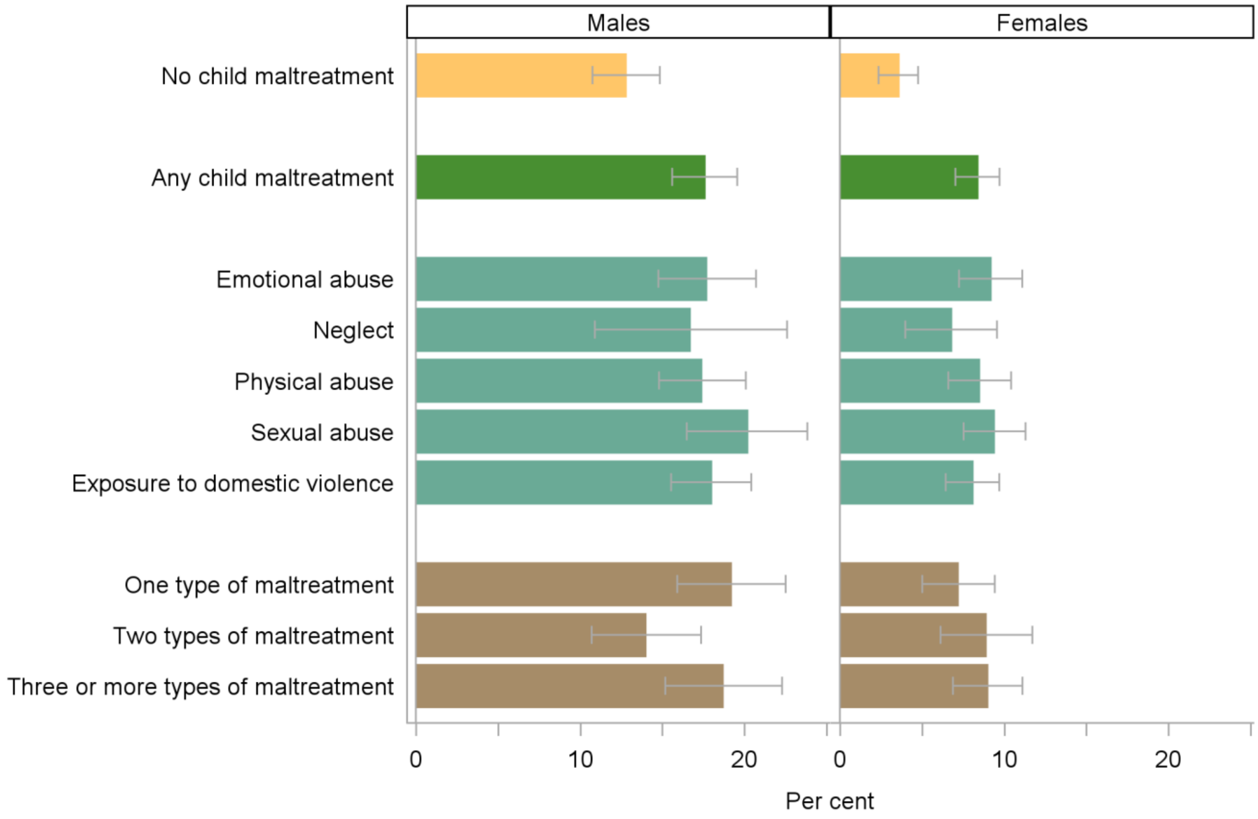

* Bar lengths represent estimated proportion of the population and error bars represent 95% CIs.

Figure 3. Proportions of adults who were classified as having cannabis dependence, by experience of child maltreatment and gender*

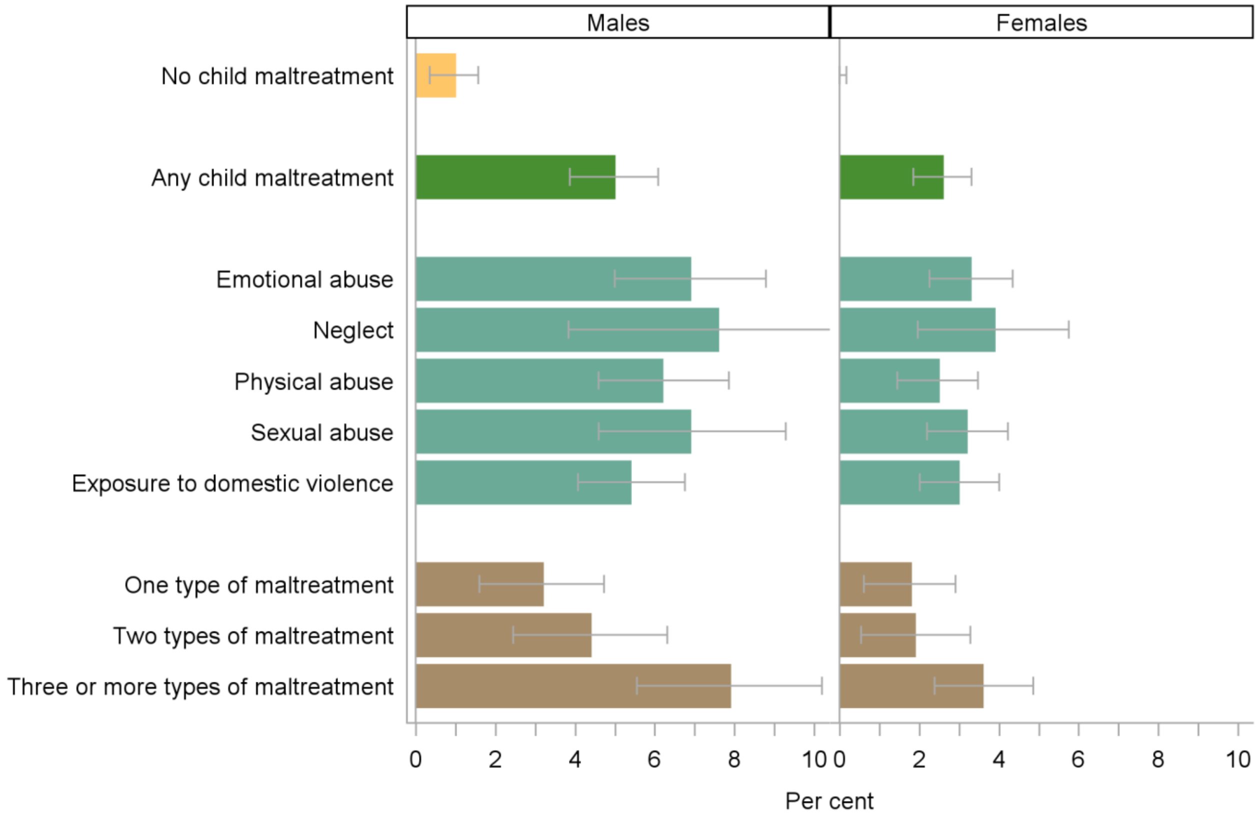

* Bar lengths represent estimated proportion of the population and error bars represent 95% CIs.

Figure 4. Proportions of adults who were classified as having obesity according to body mass index, by experience of child maltreatment and gender*

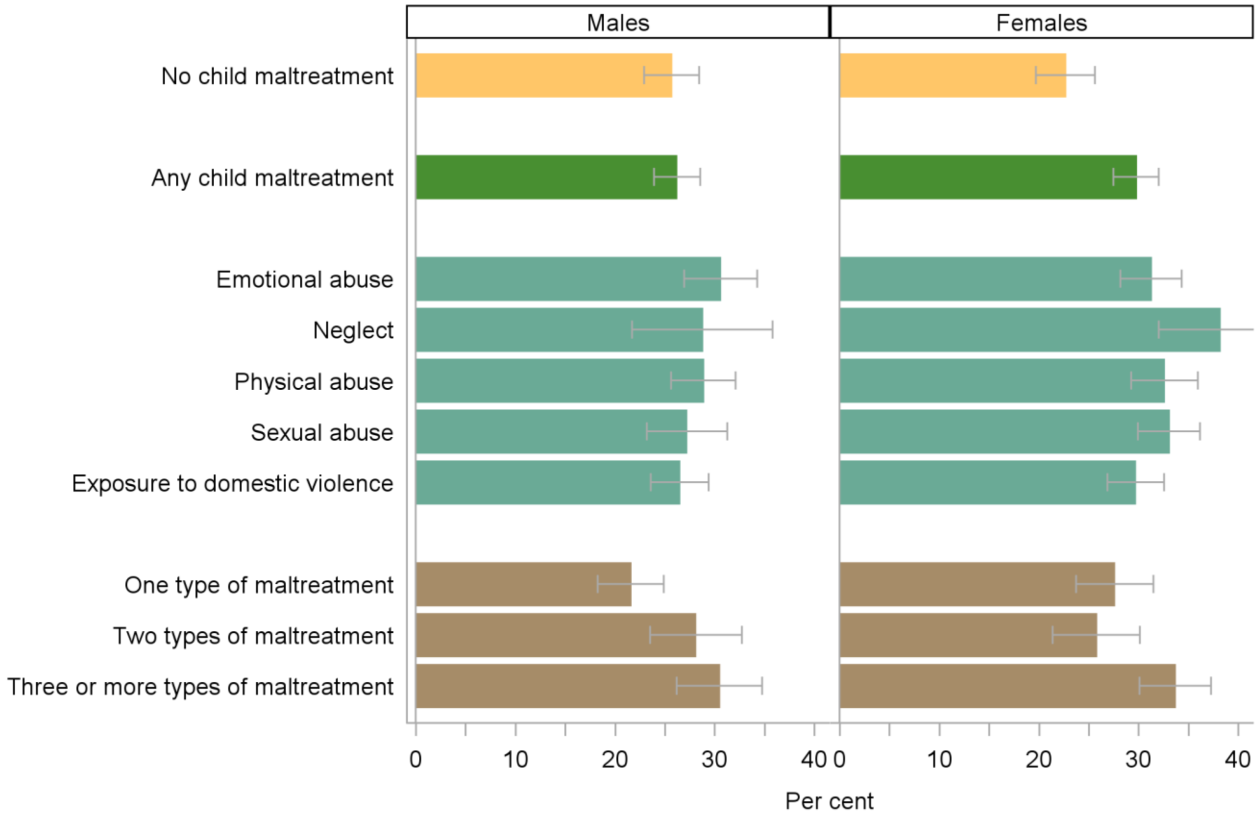

* Bar lengths represent estimated proportion of the population and error bars represent 95% CIs.

Figure 5. Proportions of adults who had self-harmed in the previous 12 months, by experience of child maltreatment and gender*

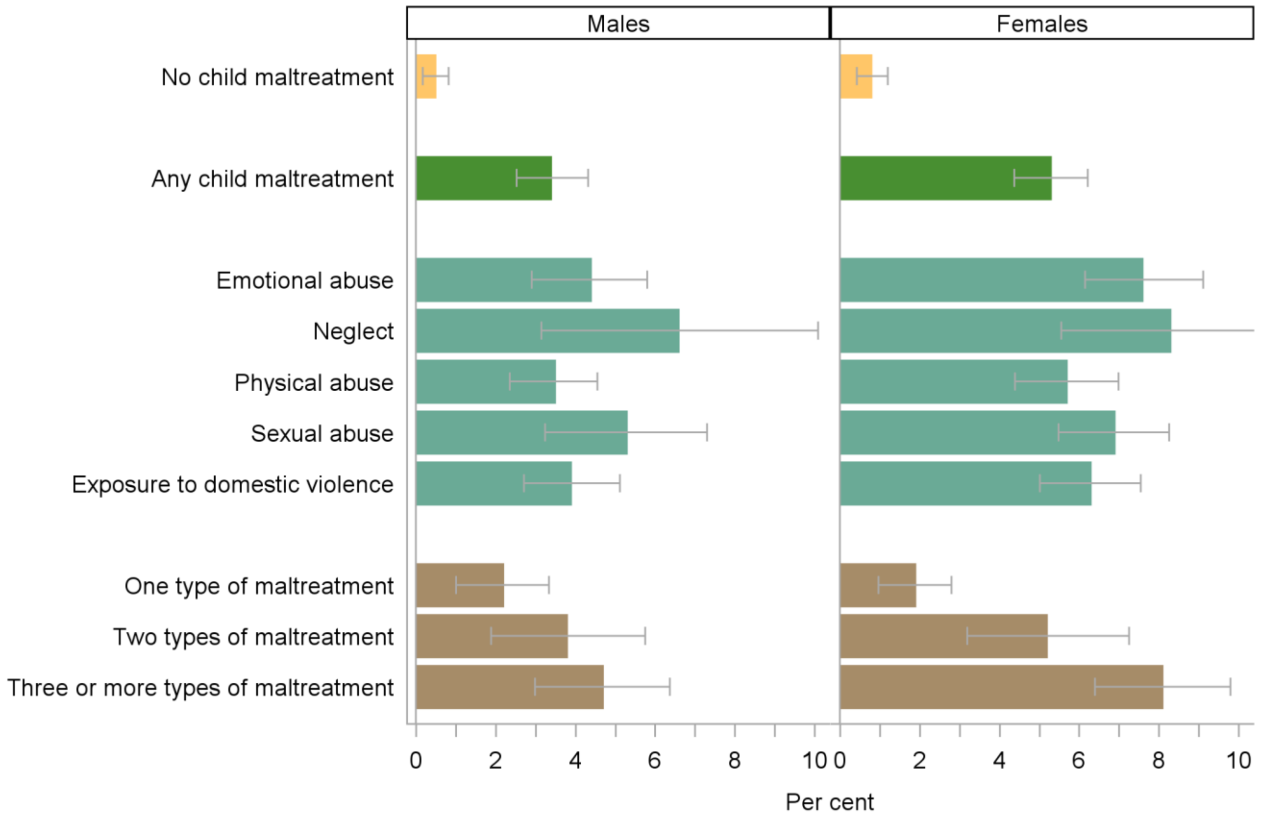

* Bar lengths represent estimated proportion of the population and error bars represent 95% CIs.

Figure 6. Proportions of adults who had attempted suicide in the previous 12 months, by experience of child maltreatment and gender*

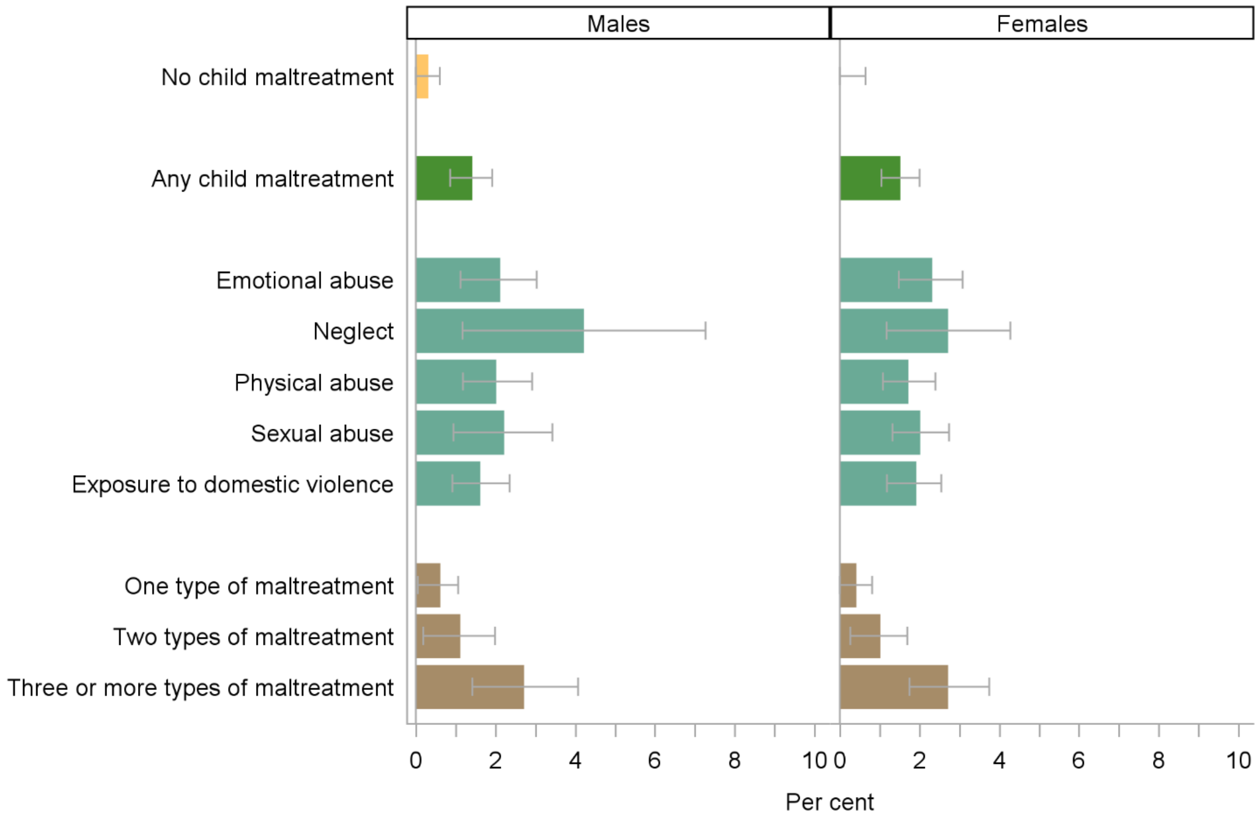

* Bar lengths represent estimated proportion of the population and error bars represent 95% CIs.
